# Effect of *Artemisia sphaerocephala* Krasch polysaccharide on the gelatinization and retrogradation of wheat starch

**DOI:** 10.1002/fsn3.1273

**Published:** 2019-11-19

**Authors:** Xiaowen Li, Junjun Li, Xiuxiu Yin, Xiaolong Wang, Tian Ren, Zhen Ma, Xiaoping Li, Xinzhong Hu

**Affiliations:** ^1^ College of Food Engineering and Nutritional Science Shaanxi Normal University Xi’an Shaanxi China

**Keywords:** *artemisia sphaerocephala* krasch polysaccharide, gelatinization, retrogradation, water retention ability, wheat starch

## Abstract

The effect of *Artemisia sphaerocephala* Krasch polysaccharide (ASKP) on the gelatinization and retrogradation of wheat starch (WS) was studied. RVA results displayed that ASKP addition increased the setback values of WS, which indicated that ASKP might promote the short‐term retrogradation of WS. DSC and XRD results demonstrated that the retrogradation percentage of WS during long‐time storage significantly decreased with the addition of ASKP, suggesting the inhibition effect of ASKP on the long‐term retrogradation of WS. As shown in TPA test, ASKP addition increased the hardness of starch gel at the beginning but decreased it after 14 days’ storage. Results of FT‐IR revealed that ASKP could promote the water retention and intermolecular hydrogen bonding formation. With the addition of ASKP, the spin–spin relaxation time measured by LF‐NMR decreased from 358.47 ms to 274.15 ms, illustrating that the WS paste containing ASKP had higher water retention ability. Leached amylose content results manifested the interaction between WS and ASKP. In summary, ASKP has potential to be a modifier on the retrogradation of starch in food processing.

## Graphical abstract

ASKP has the ability to accelerate the short‐term retrogradation of WS but decelerate the long‐term process. ASKP has potential to be a modifier on the retrogradation of starch in food processing.

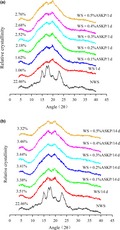

## 1 INTRODUCTION

As we all know, starch is the crucial component of starch‐based foods, and greatly affects the properties and consumer acceptance of food products (Raguzzoni, Delgadillo, & Lopes da Silva, 2016). When starches undergo a high temperature (especially above 50°C) in superfluous water, starch granules swell because of water absorption and the amylose leaches from swollen granules (Ji et al., 2016). The process, usually known as gelatinization, disrupted the ordered structure of starch and formed a hot paste (Qiu et al., 2017). When the starch paste is stored at a low temperature, it is subjected to a process called retrogradation. During the process, amylopectin and dispersed amylose molecules can form ordered structures due to recrystallization (Ji et al., 2016). In consideration of the recrystallization derived from amylose and amylopectin, there are two types of retrogradation: short‐term and long‐term types (Wang et al., 2016). Short‐term retrogradation in starch gels is more closely connected to the recombination of amylose portion, whereas long‐term retrogradation may impute to amylopectin portion (Qiu et al., 2017). The retrogradation of amylopectin was much slower than that of amylose during starch gel storage; therefore, amylopectin seemingly influenced the long‐term food storage quality (Fu, Wang, Li, Zhou, & Adhikari, 2013). Starch retrogradation is largely dependent on several factors: storage temperature, water‐to‐starch ratio, molecular structure, and addition of nonstarch components (Wang et al., 2016). Researches have shown that the addition of polysaccharide (Li, Wang, Chen, Liu, & Li, 2017), polyphenol (Zhang, Sun, Zhang, Zhu, & Tian, 2015), and amylase (Fadda, Sanguinetti, Caro, Collar, & Piga, 2014) have the ability to retard starch retrogradation.

Natural polysaccharide derived from plants or microbial sources is an important class of biomacromolecules for human health (Ren, Zhao, Nie, Yang, & Yang, 2014). Many studies reported that the addition of polysaccharides could affect starch retrogradation. For instance, Chen, Ren, Zhang, Tong, and Rashed (2015) found that the addition of pullulan could inhibit the short‐term retrogradation and long‐term retrogradation of rice starch. However, Pongsawatmanit, Chantaro, and Nishinari (2013) reported that, with the addition of xanthan, the short‐term retrogradation of tapioca starch was facilitated although opposite phenomenon was observed for the long‐term retrogradation. Another research conducted by Luo et al. (2017) manifested that the addition of inulin was able to retard amylose retrogradation, but accelerate amylopectin retrogradation of wheat starch. Moreover, Zhou, Wang, Zhang, Du, and Zhou (2008) demonstrated that the inhibitory effect of tea polysaccharide was greater than that of carboxymethyl cellulose on the retrogradation of wheat starch. These results suggested that, with different polysaccharides, different starches, the interactions between starch and polysaccharide were likely to be specific (BeMiller, 2011).

*Artemisia sphaerocephala* Krasch polysaccharide (ASKP) extracted from the outer layer of its seeds, as a water‐soluble heteropolysaccharide, has been widely used as stabilizer, thickener, and water‐holding agent in food processing in China (Guo et al., 2011). ASKP has been proved to have various biological activities, such as antitumor, hypoglycemic, hepatoprotective, and antidiabetic properties (Li et al., 2019; Ren et al., 2014; Wang, Bao, Wang, Guo, & Zhang, 2017). In addition, our research group had reported that ASKP had the potential to be an emulsifier in food production (Li, Hu, Li, & Ma, 2016). To our best knowledge, there were few reports on the effects of ASKP on starch gelatinization and retrogradation. The purpose of this research was to investigate the effects of ASKP on the gelatinization and retrogradation properties of WS and the potential mechanisms. This study is of great significance to provide guidance for the application of ASKP in improving the processing properties of starchy foods.

## 2 MATERIALS AND METHODS

### 2.1 Materials

Wheat starch with 21.3% amylose and 8.3% moisture was obtained from Shanghai Yuanye Biotechnology Co., Ltd. (Shanghai, China). *Artemisia sphaerocephala* Krasch seeds were obtained from a local market (Yulin, China). ASKP extraction was on the basis of the method of Guo et al. (2011). Briefly, ASK seeds were soaked and stirred constantly in water (1:400 w/v, 70°C) for 24 hr, and the supernatant was concentrated by rotary evaporation, then anhydrous ethanol was added to precipitate the polysaccharide, and finally, the precipitation is freeze‐dried to obtain ASKP.

### 2.2 Preparation of the mixed dispersions

ASKP‐WS mixtures were prepared according to the method described by Yang, Feng, Sun, Xu, and Zhou (2017) with some modifications. In the prepared mixtures, WS accounted for 10% and ASKP accounted for 0, 0.1, 0.2, 0.3, 0.4, and 0.5% (w/w), respectively. The suspensions were stirred evenly, followed by treatment in water bath (100°C) for one hour. Then, the cooled pastes were stored at 4°C for further studying.

### 2.3 Leached amylose content and swelling power

The content of leached amylose and swelling power of samples during heating were determined according to the method of Li et al. (2017). Firstly, the ASKP‐WS mixtures (2%) were heated in a water bath (95°C, 30 min). Then, the supernatant was stained by I_2_–KI aqueous solution (2% KI and 0.2% I_2_) and the absorbance of samples was measured at 620 nm. The precipitate was weighed and then dried to a constant weight at 105°C. Each sample was measured for three times.

### 2.4 Rapid viscosity analysis (RVA)

The pasting properties of suspensions were tested following the method of Li et al. (2017). The mixtures (2.5 g) were directly weighed in proportion into a RVA sample can and then added distilled water to 28 g. The parameters including pasting temperature (PT), peak viscosity (PV), trough viscosity (TV), final viscosity (FV), breakdown (BD = PV ‐ TV), and setback (SB = FV ‐ TV) were recorded. Each measurement was performed for three times.

### 2.5 Differential scanning calorimetry (DSC)

The influence of ASKP on the pasting and aging of WS were assessed using Thermo‐analyzer Systems (Q1000DSC + LNCS+FACS Q600SDT; TA Instruments Inc., USA) according to the method of Qiu et al. (2017). 3.0 ± 0.1 mg starch‐polysaccharide mixtures were directly weighed into test pans and then added 9 μL distilled water. The temperature was rose from 20 to 100°C at a rate of 10°C/min for gelatinization studies. The cooled samples were preserved at 4°C and then reheated under the same conditions for retrogradation analysis. The transition temperatures including the onset, peak and conclusion temperature (To, Tp, and Tc), the enthalpy of gelatinization (ΔHg), and retrogradation (ΔHr) were obtained using TA universal analysis software. Moreover, ΔHr/ΔHg x 100 was used to reflect the degree of retrogradation (%) (Raguzzoni et al., 2016).

### 2.6 Texture profile analysis (TPA)

TPA was measured based on the method reported in previous study (Li et al., 2017). The prepared pastes were sealed at 4°C for 1, 3, 7, and 14 days to allow gel formation, and returned to room temperature before the analysis. The test speed and control force were 1 mm/s and 10 g, respectively. The gels were compressed twice with 25% deformation level. The hardness and cohesiveness of the samples were recorded. Each measurement was repeated for five times.

### 2.7 X‐ray diffraction (XRD)

The ASKP‐WS gels stored at 4°C for 1 day and 14 days were orderly lyophilized, milled, and sieved (100‐mesh). XRD measurement was carried out following the method of Wang et al. (2016). Diffractograms were obtained by scanning from 4–40° (2θ) at a rate of 2°/min. Relative crystallinity was calculated using Jade 5.0 software (Materials Data Inc., Livermore). Each sample was performed for three times.

### 2.8 Fourier‐transform infrared spectroscopy (FT‐IR)

FT‐IR analysis of the samples was performed on a Fourier‐transform infrared spectrometer (Tensor27; Bruker Co., Ltd, Germany) over the range of 4000–400 per cm. Samples blended with KBr (1:100) were pressed into pellets prior to FT‐IR analysis. The amplitudes of absorbance for each sample at 1,022 per cm and 1,047 per cm were recorded.

### 2.9 Low‐field nuclear magnetic resonance (LF‐NMR)

The ASKP‐WS gels stored at 4°C for 14 days were used to perform the LF‐NMR test. The measurement was carried out using NMI20‐030H‐I (Niumag Corporation, China) according to the method of Sheng et al. (2018) with a few modifications. Parameters were set as follows, the spectral width (SW) was set to 100 KHz, the number of scans (NS) was 4, and the rapid spin‐echo pulse sequence echo time (TE) was set to 0.4 ms, P1 was 19 μs and P2 was 38 μs.

### 2.10 Statistical analysis

Data were showed as the mean ± *SD*. The mean values were analyzed with Duncan's test (*p* < .05) using SPSS 22.

## 3 RESULTS AND DISCUSSION

### 3.1 Leached amylose content and swelling power

Table 1 depicted the amount of leaching amylose of ASKP‐WS mixtures with different ASKP concentrations. The addition of ASKP could slightly increase the leached amylose content of wheat starch pastes, suggesting that ASKP could promote the leaching of amylose from wheat starch. It is possible that ASKP can interact with starch granules and facilitate the diffusion of amylose from starch granules. The result is different from some previous reports (Li et al., 2017; Sheng et al., 2018), while this phenomenon was manifested by the results of RVA experiment (Table 2).

**Table 1:** Leached amylose, swelling power, relaxation time T2 (LF‐NMR), and order degree (FT‐IR) of wheat starch with different concentrations of ASKP

Samples	Leached amylose (%)	Swelling power (g/g)	Relaxation time T2 (ms)	FT‐IR		
				DO/1d	DO/14d	ΔDO
WS	27.15 ± 0.08b	18.06 ± 0.18c	358.47a	1.179 ± 0.011d	1.427 ± 0.006abc	0.248
WS + 0.1%ASKP	28.20 ± 0.35b	19.35 ± 0.40b	355.28a	1.250 ± 0.003c	1.386 ± 0.004bc	0.136
WS + 0.2%ASKP	30.08 ± 0.53a	20.07 ± 0.31b	314.98b	1.278 ± 0.004b	1.394 ± 0.004c	0.116
WS + 0.3%ASKP	30.44 ± 1.22a	21.19 ± 0.49a	312.00b	1.312 ± 0.010a	1.406 ± 0.007ab	0.094
WS + 0.4%ASKP	30.86 ± 0.63a	21.69 ± 0.36a	293.86c	1.318 ± 0.002a	1.402 ± 0.003ab	0.084
WS + 0.5%ASKP	30.67 ± 0.52a	21.61 ± 0.22a	274.15d	1.321 ± 0.006a	1.396 ± 0.004a	0.075

Abbreviations: ASKP, *Artemisia sphaerocephala* Krasch polysaccharide; DO, order degree; ΔDO, DO/14d ‐ DO/1d; WS, wheat starch.

Means of three measurement ± *SD* values in the same column with different letters are significantly different (*p* < .05)

**Table 2:** Pasting properties (RVA) of wheat starch with different concentrations of ASKP

Samples	PT(°C)	PV(cP)	TV(cP)	FV(cP)	BD(cP)	SB(cP)
WS	64.3 ± 0.4a	910 ± 18d	725 ± 17d	1,028 ± 11e	185 ± 5d	303 ± 5e
WS + 0.1%ASKP	63.7 ± 0.7a	1,190 ± 30c	1,001 ± 24c	1,320 ± 28d	189 ± 6d	319 ± 3d
WS + 0.2%ASKP	63.7 ± 0.6a	1,233 ± 37bc	1,027 ± 16bc	1,367 ± 38cd	206 ± 2c	340 ± 6c
WS + 0.3%ASKP	63.6 ± 0.3a	1,248 ± 25abc	1,036 ± 33abc	1,405 ± 33bc	212 ± 4bc	369 ± 4b
WS + 0.4%ASKP	63.2 ± 0.4a	1,319 ± 21a	1,104 ± 27a	1,492 ± 16a	215 ± 5ab	388 ± 8a
WS + 0.5%ASKP	63.7 ± 0.1a	1,305 ± 40ab	1,076 ± 44ab	1,468 ± 35ab	229 ± 10a	392 ± 4a

Abbreviations: ASKP, *Artemisia sphaerocephala* Krasch polysaccharide; BD, Breakdown (PV‐TV); FV, Final viscosity; PT, Pasting temperature; PV, Peak viscosity; SB, Setback (FV‐TV); TV, Trough viscosity; WS, wheat starch.

Means of three measurement ± *SD* values in the same column with different letters are significantly different (Duncan's method, *p* < .05).

As shown in Table 1, the swelling power of ASKP‐WS blends increased with the increase of ASKP concentration. A plausible explanation may be that ASKP could absorb water and enhance water‐holding ability of the ASKP‐WS systems (Sheng et al., 2018).

### 3.2 Rapid viscosity analysis (RVA)

The pasting properties of WS pastes containing different amounts of ASKP were summarized in Table 2. It was well known that PV value is a parameter related to the swelling of starch granules. ASKP addition did not evidently change the PT value of WS, but it significantly increased the peak, trough, and final viscosity (910–1319 cP, 725–1104 cP, and 1028–1492 cP, respectively) with the ASKP concentration increasing. Thus, the addition of ASKP allows WS to act as an efficient thickener in foods, such as jelly, ice cream, and noodles. The result of the present research is consistent with some earlier studies (Li et al., 2017). A synergistic effect between the polysaccharide and leached amylose or swollen starch was at least responsible for the increase of viscosity (Li et al., 2017).

The breakdown (BD) value was introduced here in order to reflect the stability of the starch granules in the course of heating, and the higher the BD value, the less the granule integrity (Luo et al., 2017). Table 2 illustrated that ASKP addition caused a higher BD value. It declared that ASKP addition could reduce the stability of starch paste, and the reduction effect was strengthened with a higher ASKP concentration. The setback (SB) value is closely connected with the degree of recombination of amylose in the process of cooling and mainly reflects the degree of short‐term retrogradation (Li et al., 2017). The addition of ASKP increased the SB value from 303 to 392 cP, and a higher ASKP concentration results in a higher SB value. Previous studies have also reported that xanthan, guar gum, and β‐glucan could enhance the SB value of starch (Banchathanakij & Suphantharika, 2009; Huang, 2008; Viturawong, Achayuthakan, & Suphantharika, 2008). Our result suggested that ASKP was able to promote the short‐term retrogradation of WS, which may be related to the interactions between ASKP molecules and swollen granules or granule fragments. Another cautious speculation was that the interactions of amylose molecules were expedited due to the thickening effect of ASKP (BeMiller, 2011).

### 3.3 Differential scanning calorimetry (DSC)

Table 3 listed the gelatinization and retrogradation parameters of ASKP‐WS systems measured by DSC. The pasting temperatures (To, Tp, and Tc) were not observably changed by ASKP addition. In the majority of situations, the addition of hydrocolloids made little or no difference to gelatinization temperatures (BeMiller, 2011; Qiu et al., 2017). ASKP progressively increased the gelatinization enthalpy (ΔHg) of the starch from 11.54 J/g to 12.99 J/g. Previous studies have reported that the addition of tea polysaccharide could increase the ΔHg value of wheat starch (Zhang et al., 2015; Zhou et al., 2008). The increase in ΔHg value might be connected to the good hydrophilicity of ASKP, which can affect the water redistribution in the system. Therefore, when ASKP was added to the starch gels, more energy is needed for gelatinization (Yang et al., 2017).

**Table 3:** DSC measurement for gelatinization and retrogradation properties of wheat starch with different concentrations of ASKP

Samples	To (°C)	Tp (°C)	Tc (°C)	ΔHg (J/g)	ΔHr_1_ (J/g)	R_1_(%)	ΔHr_14_ (J/g)	R_14_(%)
WS	58.95 ± 0.11a	62.07 ± 0.21a	68.94 ± 0.04a	11.54 ± 0.08d	5.07 ± 0.04e	43.93	6.80 ± 0.03e	58.93
WS + 0.1%ASKP	58.70 ± 0.03ab	62.29 ± 0.08a	69.09 ± 0.04a	12.47 ± 0.11c	5.54 ± 0.04d	44.43	7.08 ± 0.04d	56.78
WS + 0.2%ASKP	58.51 ± 0.18b	61.94 ± 0.13a	69.03 ± 0.25a	12.62 ± 0.05bc	5.66 ± 0.02c	44.85	7.18 ± 0.04c	56.89
WS + 0.3%ASKP	58.96 ± 0.21a	62.23 ± 0.11a	69.08 ± 0.21a	12.80 ± 0.04ab	5.78 ± 0.02b	45.16	7.30 ± 0.04b	57.03
WS + 0.4%ASKP	58.67 ± 0.18ab	62.02 ± 0.04a	69.04 ± 0.25a	12.90 ± 0.12a	5.87 ± 0.03ab	45.50	7.38 ± 0.04a	57.21
WS + 0.5%ASKP	58.59 ± 0.02ab	62.00 ± 0.16a	69.04 ± 0.28a	12.99 ± 0.08a	5.92 ± 0.08a	45.57	7.44 ± 0.01a	57.27

Abbreviations: ASKP, *Artemisia sphaerocephala* Krasch polysaccharide; Tc, Conclusion temperature; To, Onset temperature; Tp, Peak temperature; ΔHg, Enthalpy of gelatinization; ΔHr_i_, Enthalpy of retrogradation at i ^th^ day; R_i_, Percentage of retrogradation at i ^th^ day (ΔHr_i_/ΔHg)*100; WS, wheat starch.

Means of three measurement ± *SD* values in the same column with different letters are significantly different (Duncan's method, *p* < .05).

Enthalpy of retrogradation (ΔHr) mainly represents the degree of recrystallization for amylopectin (Luo et al., 2017). Therefore, as reported by previous studies, the ΔHr values of samples tended to increase with storage period (Chen et al., 2015; Li et al., 2017). The percentage of retrogradation (R_i_) reflects the ratio of ΔHr to ΔHg, and the lower the *R* value, the stronger the suppression on retrogradation of starch (Luo et al., 2017). After 1 day at 4°C, the *R*_1_ value of WS increased from 43.91% to 45.54% as the content of ASKP was increased from 0% to 0.5%. It was consistent with RVA results. After 14 days, the R_14_ value decreased from 58.95% to 56.76%. The ΔR (= R_14_ ‐ R_1_) reflected the degree of retrogradation during the 13‐day storage, and decreased from 15.00% to 11.70% with the changes in polysaccharide concentration. These results indicated that ASKP could accelerate the short‐term retrogradation and impede the long‐term process of WS, which were in accordance with previous literature regarding to retrogradation of starch‐hydrocolloid blends (Pongsawatmanit et al., 2013). It was hypothesized that ASKP could prevent the reassociation of amylopectin molecules through interactions with amylopectin (Yoshimura, Takaya, & Nishinari, 1996).

### 3.4 Texture profile analysis (TPA)

**Figure 1:**
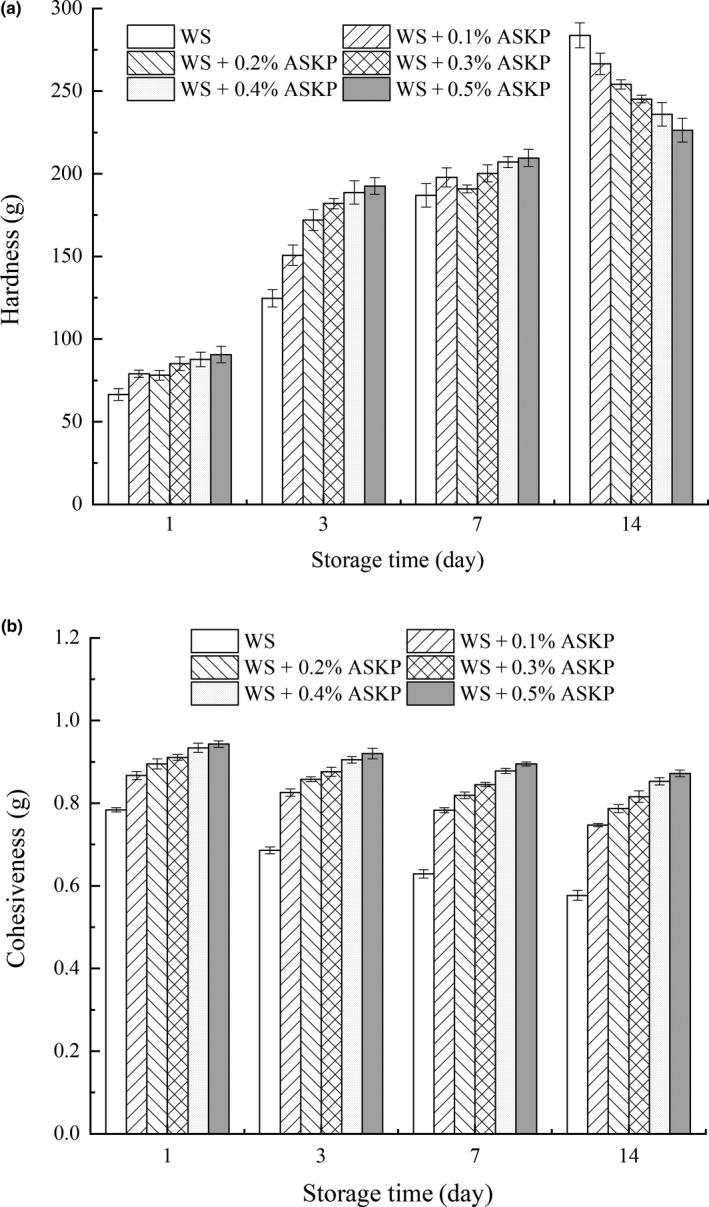
The hardness (a) and cohesiveness (b) of wheat starch with different concentrations of ASKP during storage. ASKP, *Artemisia sphaerocephala* Krasch polysaccharide; WS, wheat starch

The hardness of ASKP‐WS gels determined by TPA was illustrated in Figure 1a. Hardness of starch gel can be applied to estimate the degree of retrogradation because it is strongly relevant to retrogradation (Tian et al., 2009). When the ASKP‐WS gels were stored at 4°C, the hardness of all tested samples increased with the storage time. The hardness of gels containing ASKP was larger than without ASKP at 1 and 3 days, which indicates that ASKP has the potential to enhance the hardness of WS gel during short‐term storage. This was in agreement with the SB values in RVA results (Table 2). The hardness of tested samples with and without ASKP was inclined to be equal in the seventh day. Then, the gels containing ASKP became softer than the starch‐only samples at 14 days, and the hardness of tested samples decreased with the increasing of ASKP concentration. The results were in keeping with the R_14_ values in DSC results (Table 3). Hence, ASKP could be applied in soup, noodles, jelly, and other starchy food according to different firmness demands and different storage time. The aforementioned phenomenon suggested that ASKP could strengthen the short‐term retrogradation of WS, while retard the long‐term retrogradation. These are in good agreement with reported research that xanthan or gum arabic could increase markedly initial gel hardness, but decrease hardness during longer storage (Alloncle & Doublier, 1991). The change in the gel firmness may be ascribed to the competition of available water between the molecules of starch and ASKP and interactions between ASKP and WS granules.

Cohesiveness is a parameter related to intramolecular interactions. During the same storage time, the cohesiveness of the tested gel was increased with the addition of ASKP (Figure 1b), indicating that stronger intramolecular interactions formed in the gels containing ASKP. However, with time going on, the cohesiveness of all gels decreased slightly. Zhang et al. (2015) also pointed out that tea polysaccharide has ability to aggrandize the cohesiveness of the wheat starch gel.

### 3.5 X‐ray diffraction patterns (XRD)

**Figure 2:**
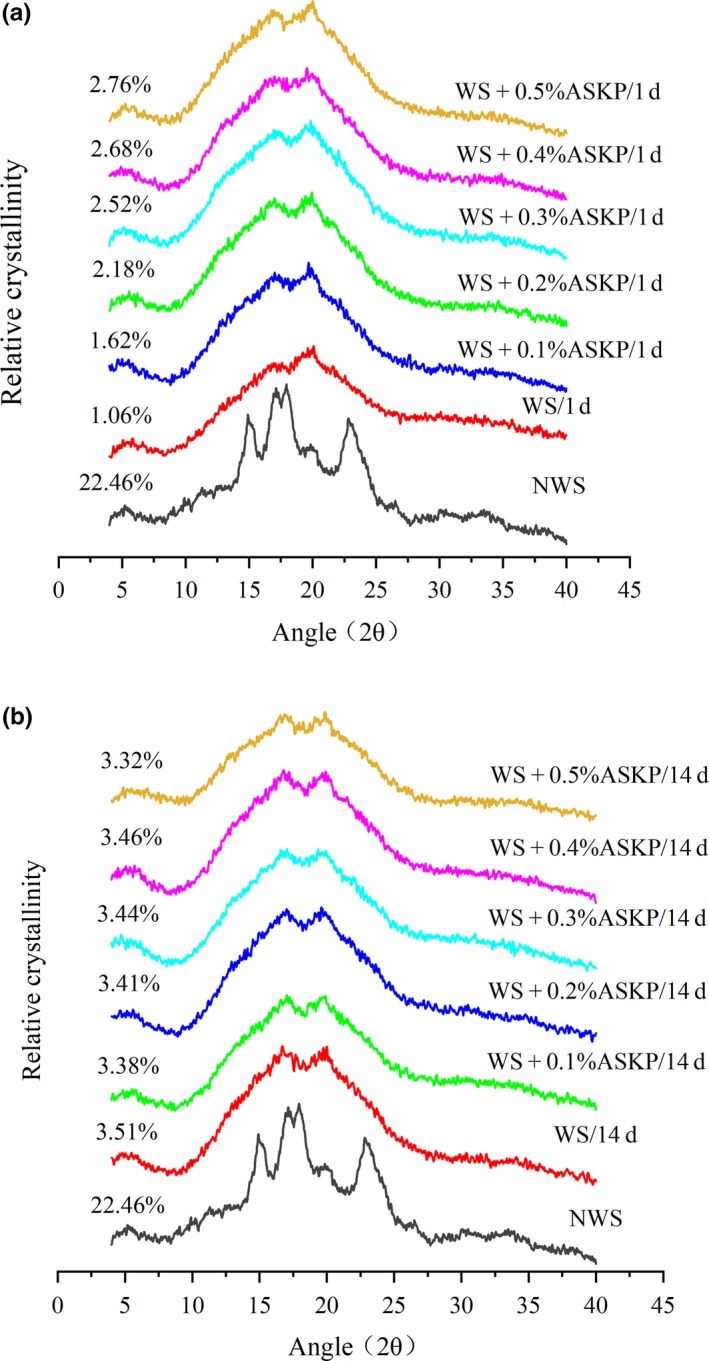
X‐ray diffraction patterns of native wheat starch and gelatinized wheat starch paste containing various ratios of ASKP after 1 days’ (a) and 14 days’ (b) storage at 4°C. ASKP, *Artemisia sphaerocephala* Krasch polysaccharide; WS, wheat starch

XRD experiment was adopted in order to further explore the influence of ASKP on the aging of WS (Nagano, Tamaki, & Funami, 2008). XRD patterns of WS and ASKP‐WS mixtures were depicted in Figure 2.

As previous literature reported, there are four crystal types for XRD patterns of plant starch. 2θ at around 15, 17, 18, 20, and 23 are A‐type crystal, 5.5, 17, 22, and 24 are B‐type crystal, and 7.8, 13.5, and 20.7 are V‐type crystal. The mixture of A‐type and B‐type is defined as C‐type (Luo et al., 2017). Native wheat starch belonged to typical A‐type XRD pattern due to obvious diffraction peaks at 15.2, 17.0, 18.2, and 23.1 (Figure 2), which is consistent with previous studies (Li et al., 2017; Luo et al., 2017). After gelatinization and retrogradation, the characteristic peaks of all samples were located at about 5.5, 16.8, 19.9 (Figure 2), demonstrating that the WS formed a C‐type crystal.

As shown in Figure 2, since the presence of ASKP made no significant difference on diffraction peak location, ASKP could not change starch crystal type. With the increasing in ASKP concentration, the characteristic peak intensity of C‐type diffraction pattern was strengthened, so ASKP addition was able to expedite the recrystallization of starch (especially amylose) (Figure 2a). However, when the starch gel was stored for 14 days, peak intensity of C‐type structure tended to be weaker with the increased amounts of ASKP (Figure 2b), implying the inhibiting effect of ASKP on the reaggregation of starch molecule (particularly amylopectin).

When the concentration of ASKP increased from 0% to 0.5%, the RC (relative crystallinity) value of ASKP‐WS stored for 1 day increased from 1.06% to 2.76%, while in contrast, the RC value of the samples stored for 14 days decreased from 3.51% to 3.32% (Figure 2). ΔRC represented the increase of relative crystallinity. A lower ΔRC value of WS was observed during storage from 1 day to 14 days. Overall, these phenomena confirmed that ASKP addition could promote short‐term retrogradation but hinder long‐term retrogradation of WS, which was in line with DSC result (Table 3).

### 3.6 Fourier‐transform infrared spectroscopy (FT‐IR)

**Figure 3:**
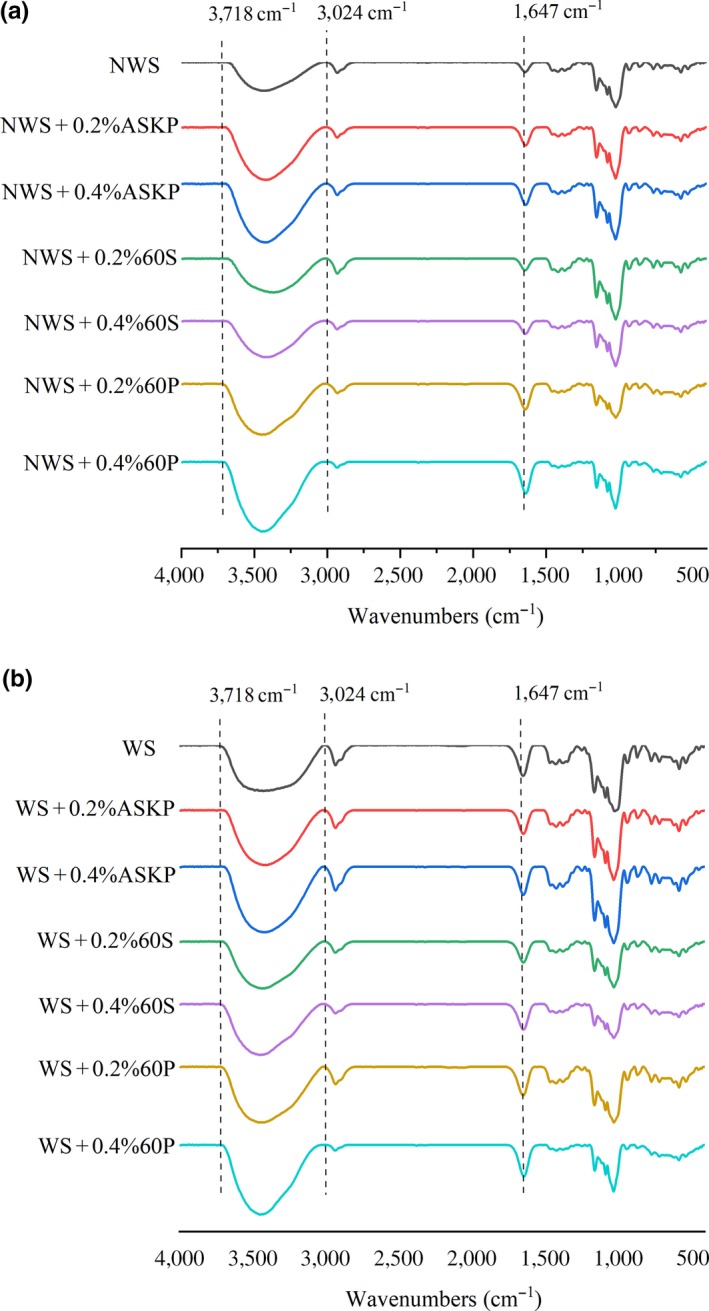
FT‐IR spectra of the native WS containing various ratios of ASKP (a) and gelatinized WS paste containing various ratios of ASKP after 14 days’ (b) storage at 4°C. ASKP, *Artemisia sphaerocephala* Krasch polysaccharide; WS, wheat starch after gelatinization; NWS, native wheat starch before gelatinization

In order to detect changes in the short‐range order of WS with the addition of ASKP, infrared spectroscopy was conducted both before and after gelation. Figure 3a displayed the FT‐IR spectra in the range of 400–4,000 per cm of ASKP‐WS mixtures before gelation, and Figure 3b depicted the samples suffered gel treatment.

The FT‐IR spectra of all tested samples were similar, which implied that ASKP addition made no significant difference in chemical groups of WS. However, the absorption intensity of ASKP‐WS around 1,640–1,650 per cm and 3,100–3,700 per cm was stronger than that of native starch, suggesting that more hydrogen bond formed in ASKP‐WS mixtures. The absorption peak at 1,022 per cm was connected with vibrational modes within the amorphous phase of starch. At the same time, ordered structures of starch were described by the mode at 1,047 per cm. The ratio of intensity at 1047/1022 per cm (DO) was adopted to describe the degree of order in starches (López‐Rubio, Flanagan, Shrestha, Gidley, & Gilbert, 2008; Zeng et al., 2015).

With the concentration of ASKP increasing from 0% to 0.5%, the DO values of ASKP‐WS stored for 1 day increased from 1.179 to 1.321, while the values of same samples stored for 14 days decreased from 1.427 to 1.394 (Table 1). This trend was consistent with relative crystallinity (Figure 2), manifesting that ASKP addition could promote short‐term retrogradation but hinder long‐term retrogradation of WS.

### 3.7 Low‐field nuclear magnetic resonance (LF‐NMR)

LF‐NMR was used to detect water changes and evaluate the water‐holding capacity in food materials (Sheng et al., 2018). The spin–spin relaxation time (T2) was used to describe the changes in water mobility. In starch paste system, a lower T2 value means lower water mobility, indicating the tighter association between water molecules and starch molecules (Li et al., 2015). Table 1 displays the T2 values of ASKP‐WS systems with different concentrations of ASKP during long‐term storage. Apparently, T2 values (358.47–274.15 ms) decreased with the ASKP concentration increased, indicating that ASKP could constrain the free motion of water molecules. ASKP is a kind of hydrophilic polysaccharide and has good water‐holding ability. Therefore, the changes of T2 might be related to the hydrogen bonds formed by ASKP and WS molecules, which weakened the interaction between starch and water and consequently restrained the long‐term retrogradation of WS. This was in keeping with the FT‐IR results. Similar reports have been disclosed by Charoenrein, Tatirat, Rengsutthi, and Thongngam (2011) and Sheng et al. (2018) that konjac glucomannan and pullulan were able to promote the water‐holding capacity of starch paste. In short, ASKP addition could motivate water retention ability of WS paste and block long‐term retrogradation of WS.

## 4 CONCLUSIONS

Based on the results, ASKP has the ability to accelerate the short‐term retrogradation of WS but decelerate the long‐term process. During gelatinization, the peak viscosity, breakdown, setback, and ΔHg values of WS increased with the addition of ASKP. During the short‐term storage, ASKP addition increased retrogradation enthalpy, gel hardness, and relative crystallinity of WS. Nevertheless, ASKP addition decreased retrogradation enthalpy, gel hardness, and relative crystallinity of WS during the long‐term storage. The addition of ASKP enhanced the hydrogen bonding effect and restricted the water mobility in a dose‐dependent manner. The observed phenomena might be attributed to changes in starch granule swelling, interaction between hydrocolloid molecule with leached starch polymer molecules, competition between hydrocolloid and amylose or amylopectin for available water molecules. Overall, these findings manifested that the ASKP could be promising alternatives for improving functionalities of starch products.

## CONFLICT OF INTERESTS

The authors declare that there is no conflict of interests.

## ETHICAL STATEMENT

This study does not involve any human or animal testing.

## References

[fsn31273-bib-0001] Alloncle, M. , & Doublier, J. L. (1991). Viscoelastic properties of maize starch/hydrocolloid pastes and gels. Food Hydrocolloids, 5, 455–467. 10.1016/S0268-005X(09)80104-5

[fsn31273-bib-0002] Banchathanakij, R. , & Suphantharika, M. (2009). Effect of different β‐glucans on the gelatinisation and retrogradation of rice starch. Food Chemistry, 114, 5–14. 10.1016/j.foodchem.2008.09.016

[fsn31273-bib-0003] BeMiller, J. N. (2011). Pasting, paste, and gel properties of starch‐hydrocolloid combinations. Carbohydrate Polymers, 86, 386–423. 10.1016/j.carbpol.2011.05.064

[fsn31273-bib-0004] Charoenrein, S. , Tatirat, O. , Rengsutthi, K. , & Thongngam, M. (2011). Effect of konjac glucomannan on syneresis, textural properties and the microstructure of frozen rice starch gels. Carbohydrate Polymers, 83, 291–296. 10.1016/j.carbpol.2010.07.056

[fsn31273-bib-0005] Chen, L. , Ren, F. , Zhang, Z. , Tong, Q. , & Rashed, M. M. A. (2015). Effect of pullulan on the short‐term and long‐term retrogradation of rice starch. Carbohydrate Polymers, 115, 415–421. 10.1016/j.carbpol.2014.09.006 25439913

[fsn31273-bib-0006] Fadda, C. , Sanguinetti, A. M. , Del Caro, A. , Collar, C. , & Piga, A. (2014). Bread staling: Updating the view. Comprehensive Reviews in Food Science & Food Safety, 13, 473–492. 10.1111/1541-4337.12064 33412702

[fsn31273-bib-0007] Fu, Z. Q. , Wang, L. J. , Li, D. , Zhou, Y. G. , & Adhikari, B. (2013). The effect of partial gelatinization of corn starch on its retrogradation. Carbohydrate Polymers, 97, 512–517. 10.1016/j.carbpol.2013.04.089 23911478

[fsn31273-bib-0008] Guo, Q. , Cui, S. W. , Wang, Q. I. , Hu, X. , Guo, Q. , Kang, J. I. , & Yada, R. (2011). Extraction, fractionation and physicochemical characterization of water‐soluble polysaccharides from Artemisia sphaerocephala Krasch seed. Carbohydrate Polymers, 86, 831–836. 10.1016/j.carbpol.2011.05.034

[fsn31273-bib-0009] Huang, C. C. (2008). Physicochemical, pasting and thermal properties of tuber starches as modified by guar gum and locust bean gum. International Journal of Food Science and Technology, 44, 50–57.

[fsn31273-bib-0010] Ji, N. , Liu, C. , Zhang, S. , Yu, J. , Xiong, L. , & Sun, Q. (2016). Effects of chitin nano‐whiskers on the gelatinization and retrogradation of maize and potato starches. Food Chemistry, 214, 543–549. 10.1016/j.foodchem.2016.07.113 27507508

[fsn31273-bib-0011] Li, J. , Hu, X. , Li, X. , & Ma, Z. (2016). Effects of acetylation on the emulsifying properties of Artemisia sphaerocephala Krasch polysaccharide. Carbohydrate Polymers, 144, 531–540. 10.1016/j.carbpol.2016.02.039 27083845

[fsn31273-bib-0012] Li, J. , Zhao, H. , Hu, X. , Shi, J. , Shao, D. , & Jin, M. (2019). Antidiabetic effects of different polysaccharide fractions from Artemisia sphaerocephala Krasch seeds in db/db mice. Food Hydrocolloids, 91, 1–9. 10.1016/j.foodhyd.2019.01.002

[fsn31273-bib-0013] Li, Q. Q. , Wang, Y. S. , Chen, H. H. , Liu, S. , & Li, M. (2017). Retardant effect of sodium alginate on the retrogradation properties of normal cornstarch and anti‐retrogradation mechanism. Food Hydrocolloids, 69, 1–9. 10.1016/j.foodhyd.2017.01.016

[fsn31273-bib-0014] Li, Z. , Liu, W. , Gu, Z. , Li, C. , Yan, H. , & Cheng, L. (2015). The effect of starch concentration on the gelatinization and liquefaction of corn starch. Food Hydrocolloids, 48, 189–196. 10.1016/j.foodhyd.2015.02.030

[fsn31273-bib-0015] López‐Rubio, A. , Flanagan, B. , Shrestha, A. , Gidley, M. , & Gilbert, E. (2008). Molecular rearrangement of starch during in vitro digestion: Toward a better understanding of enzyme resistant starch formation in processed starches. Biomacromolecules, 9, 1951–1958. 10.1021/bm800213h 18529077

[fsn31273-bib-0016] Luo, D. , Li, Y. , Xu, B. , Ren, G. , Li, P. , Li, X. , … Liu, J. (2017). Effects of inulin with different degree of polymerization on gelatinization and retrogradation of wheat starch. Food Chemistry, 229, 35–43. 10.1016/j.foodchem.2017.02.058 28372184

[fsn31273-bib-0017] Nagano, T. , Tamaki, E. , & Funami, T. (2008). Influence of guar gum on granule morphologies and rheological properties of maize starch. Carbohydrate Polymers, 72, 95–101. 10.1016/j.carbpol.2007.07.028

[fsn31273-bib-0018] Pongsawatmanit, R. , Chantaro, P. , & Nishinari, K. (2013). Thermal and rheological properties of tapioca starch gels with and without xanthan gum under cold storage. Journal of Food Engineering, 117, 333–341. 10.1016/j.jfoodeng.2013.03.010

[fsn31273-bib-0019] Qiu, S. , Yadav, M. P. , Zhu, Q. , Chen, H. , Liu, Y. , & Yin, L. (2017). The addition of corn fiber gum improves the long‐term stability and retrogradation properties of corn starch. Journal of Cereal Science, 76, 92–98. 10.1016/j.jcs.2017.04.012

[fsn31273-bib-0020] Raguzzoni, J. C. , Delgadillo, I. , & Lopes da Silva, J. A. (2016). Influence of a cationic polysaccharide on starch functionality. Carbohydrate Polymers, 150, 369–377. 10.1016/j.carbpol.2016.05.024 27312647

[fsn31273-bib-0021] Ren, D. , Zhao, Y. , Nie, Y. , Yang, N. , & Yang, X. (2014). Hypoglycemic and hepatoprotective effects of polysaccharides from Artemisia sphaerocephala Krasch seeds. International Journal of Biological Macromolecules, 69, 296–306. 10.1016/j.ijbiomac.2014.05.064 24887549

[fsn31273-bib-0022] Sheng, L. , Li, P. , Wu, H. , Liu, Y. , Han, K. E. , Gouda, M. , … Jin, Y. (2018). Tapioca starch‐pullulan interaction during gelation and retrogradation. LWT‐Food Science and Technology, 96, 432–438. 10.1016/j.lwt.2018.05.064

[fsn31273-bib-0023] Tian, Y. , Li, Y. , Manthey, F. A. , Xu, X. , Jin, Z. , & Deng, L. (2009). Influence of β‐cyclodextrin on the short‐term retrogradation of rice starch. Food Chemistry, 116, 54–58. 10.1016/j.foodchem.2009.02.003

[fsn31273-bib-0024] Viturawong, Y. , Achayuthakan, P. , & Suphantharika, M. (2008). Gelatinization and rheological properties of rice starch/xanthan mixtures: Effects of molecular weight of xanthan and different salts. Food Chemistry, 111, 106–114. 10.1016/j.foodchem.2008.03.041

[fsn31273-bib-0025] Wang, J. , Bao, A. , Wang, Q. , Guo, H. , & Zhang, J. (2017). Sulfation can enhance antitumor activities of artemisia sphaerocephala polysaccharide in vitro and vivo. International Journal of Biological Macromolecules, 107, 502–511. 28893683 10.1016/j.ijbiomac.2017.09.018

[fsn31273-bib-0026] Wang, L. , Xu, J. , Fan, X. , Wang, Q. , Wang, P. , Zhang, Y. , … Yu, Y. (2016). Effect of disaccharides of different composition and linkage on corn and waxy corn starch retrogradation. Food Hydrocolloids, 61, 531–536. 10.1016/j.foodhyd.2016.06.010

[fsn31273-bib-0027] Yang, X. , Feng, M. Q. , Sun, J. , Xu, X. L. , & Zhou, G. H. (2017). The influence of flaxseed gum on the retrogradation of maize starch. International Journal of Food Science & Technology, 52(12), 2654–2660. 10.1111/ijfs.13554

[fsn31273-bib-0028] Yoshimura, M. , Takaya, T. , & Nishinari, K. (1996). Effects of konjac‐glucomannan on the gelatinization and retrogradation of corn starch as determined by rheology and differential scanning calorimetry. Journal of Agricultural & Food Chemistry, 44, 2970–2976. 10.1021/jf960221h

[fsn31273-bib-0029] Zeng, S. , Wu, X. , Lin, S. , Zeng, H. , Lu, X. U. , Zhang, Y. I. , & Zheng, B. (2015). Structural characteristics and physicochemical properties of lotus seed resistant starch prepared by different methods. Food Chemistry, 186, 213–222. 10.1016/j.foodchem.2015.03.143 25976813

[fsn31273-bib-0030] Zhang, H. , Sun, B. , Zhang, S. , Zhu, Y. , & Tian, Y. (2015). Inhibition of wheat starch retrogradation by tea derivatives. Carbohydrate Polymers, 134, 413–417. 10.1016/j.carbpol.2015.08.018 26428142

[fsn31273-bib-0031] Zhou, Y. , Wang, D. , Zhang, L. , Du, X. , & Zhou, X. (2008). Effect of polysaccharides on gelatinization and retrogradation of wheat starch. Food Hydrocolloids, 22, 505–512. 10.1016/j.foodhyd.2007.01.010

